# Transfer of attunement in length perception by dynamic touch

**DOI:** 10.3758/s13414-015-0872-y

**Published:** 2015-03-26

**Authors:** Simon de Vries, Rob Withagen, Frank T. J. M. Zaal

**Affiliations:** Center for Human Movement Sciences, University Medical Center Groningen, University of Groningen, P.O. Box 196, 9700 AD Groningen, The Netherlands

**Keywords:** Dynamic touch, Individual differences, Perceptual learning, Transfer

## Abstract

Earlier studies have revealed that the calibration of an action sometimes transfers in a functionally specific way—the calibration of one action transfers to other actions that serve the same goal, even when they are performed with different anatomical structures. In the present study, we tested whether attunement (the process by which perceivers learn to detect a more useful, specifying, informational pattern) follows such a functional organization. Participants were trained to perceive the length of rods by dynamic touch with one of their effectors. It was found that training the right hand resulted in an attunement to a specifying variable with both hands, but not with the feet. Training the other limbs did not result in attunement. However, substantial individual differences were found. The implications of the results are explored for theories on the organization of perceptual learning and discussions on individual differences in perception.

The study of transfer of calibration in perception and action has a long history. Calibration refers to the process that establishes an appropriate scaling of information to perception or action (Bingham & Pagano, 1998; Jacobs & Michaels, 2006; Wagman & Abney, 2013; Withagen & Michaels, 2004). Ever since the seminal work of Helmholtz in the late 19th century, prism studies have been used to induce a calibration (Cohen, 1967; Hamilton, 1964). Wearing prisms yields a shift in the visual field and thus requires a rescaling of the optical information to the movement. In the second half of the previous century, several studies have tested whether such a recalibration is confined to the exposed limb or transfers to the unexposed limb. Under certain conditions, a transfer of calibration has been found (Cohen, 1967; Hamilton, 1964).

Inspired by these findings, Rieser, Pick, Ashmead, and Garing (1995) addressed the question of the organization of calibration. They distinguished the anatomical model from the functional model. The former holds that calibration is confined to the trained anatomical structure, implying that the calibration manifests itself in any behaviour that is performed by this structure. The functional model, on the other hand, states that the calibration of an action transfers to all actions that serve the same goal, irrespective of the bodily structures that are participating in performing the action. This model is based on the principle of *motor equivalence* (Hebb, 1949; Lashley, 1930)—the same goal can be achieved in different ways in which different anatomical structures are involved. For example, a human-being can locomote by means of walking, side-stepping, crawling, and other movements. The functional model would predict that calibration of one means of achieving a goal would transfer to other means of achieving the same goal, regardless of the anatomical structures involved. During the past decades, the functional model has been put to several tests. Although functionally specific transfer has been observed (Rieser et al. 1995; Stephen & Hajnal, 2011; Withagen & Michaels, 2002, 2004), not all studies found it (Durgin, Fox, & Kim, 2003; Bingham, Pan, & Mon-Williams, 2014; Martin, Keating, Goodkin, Bastian, & Thach, 1996; Withagen & Michaels, 2007). Bingham et al. (2014) explained this discrepancy with his “mapping theory.” According to this theory, calibration can be functionally specific, but it also can be confined to a specific limb. “[T]he Mapping Theory predicts that limb specific calibration should be possible because the units are embodied and anatomy contributes to their scaling” (Bingham et al., 2014, p. 61).

As argued by ecological psychologists, calibration is *one* means by which perception and action can change. Perception and action should not only be appropriately scaled to information but also should be based on a useful informational variable (Jacobs & Michaels, 2006; Stephen & Arzamarski, & Michaels, 2010; Withagen & Michaels, 2005). Indeed, perception and action can improve by learning to rely on the more useful information (Gibson & Gibson, 1955). This process has been referred to as the *education of attention* or *attunement* and has been observed in different paradigms (Fajen, 2005; Jacobs & Michaels, 2007; Withagen & van der Kamp, 2010). Although the process of attunement has been extensively studied in the past decades, the organization of attunement, to our knowledge, has not been addressed in the literature. Does attunement follow a functional organization or is it anatomically specific? In the present study, we examine this by using the paradigm of length perception by dynamic touch.

Dynamic touch refers to the perceptual modality by which perceivers can feel several properties of wielded objects. For example, by holding a rod in one of the hands and wielding it, one can feel, among other things, its weight, length, and shape (Cabe, 2010; Turvey & Carello, 1995; Wagman & Carello, 2001). The reason for testing transfer of attunement in the domain of dynamic touch is threefold. First, earlier studies have revealed that when provided with feedback, perceivers can learn to rely on more useful, or even specifying, mechanical variables in their estimation of length (Arzamarski, Isenhower, Kay, Turvey, & Michaels, 2010; Menger & Withagen, 2009; Michaels, Arzamarski, Isenhower, & Jacobs, 2008; Wagman, Shockley, Riley, & Turvey, 2001; Withagen & Michaels, 2005). Novice perceivers have been found to detect the major principal moment of inertia (*I*_*1*_) and/or static moment (*M*) (Solomon and Turvey, 1988; Kingma, van de Langenberg, & Beek, 2004; Withagen & van Wermeskeren, 2009). These variables relate ambiguously to the length of rods; they are a function of the rod’s length, diameter, and material density. Thus, equal length rods made of different material can vary in *I*_*1*_ and *M*; and rods that differ in length can have the same *I*_*1*_ and *M*. Being related ambiguously to the to-be-perceived property (i.e., actual length), these variables have been referred to as nonspecifying variables (Withagen & Michaels, 2005). Importantly, when provided with feedback, perceivers have been found to attune to mechanical variables that are specific to the length of homogeneous rods. An instance of such a specifying mechanical variable is the ratio of *I*_*1*_ to *M*.

$$ \frac{I_1}{M}=\frac{\frac{1}{3}\ast m\ast {L}^2}{m\ast L/2}=\frac{2}{3}L $$

where *m* is mass and *L* is length. Because the mass cancels out, the ratio of *I*_*1*_ to *M* relates one-to-one to the length of homogeneous rods (as any other ratio of two moments of mass distribution). Hence, if perceivers initially detect, say *I*_*1*_, and then learn to detect a specifying variable, their perception of length improves—i.e., rods that are of equal length can first be perceived as being different in length, but when a specifying variable is attuned to, the rods are perceived as being of the same length. Perceivers have been found to be capable of learning to rely on a specifying mechanical variable (Menger & Withagen, 2009; Michaels et al., 2008; Withagen & Michaels, 2005), although large individual differences in this capability have been reported (Rop & Withagen, 2014; Withagen & Caljouw, 2011; Withagen & van Wermeskerken, 2009).

A second reason for using the paradigm of dynamic touch for our study of transfer of attunement is that the above-mentioned, nonspecifying mechanical variables have been found to be detectable with different anatomical structures. As Gibson (1966) argued in his seminal book, *The Senses Considered as Perceptual Systems,* perceptual systems should not be conceived of as channels of sensation identified by a particular piece of anatomy. Indeed, many bodily parts are involved in the detection of information. Moreover, the same informational pattern can be detected by means of different anatomical structures (Bongers & Michaels, 2008; Oudejans, Michaels, Bakker, & Davids, 1999). This phenomenon has been referred to as the *multiple realizability of functions* and has been studied in the domain of dynamic touch. Carello, Fitzpatrick, Domaniewicz, Chan, and Turvey (1992) stated that the haptic perceptual system is *softly assembled,* which means that it is a functional organization that is temporarily assembled over different anatomical structures (see also Wagman & Hajnal, 2014a, 2014b). For instance, in perceiving length, one can detect mechanical variable(s) with either the right hand or the left hand, and the wielding can be around the wrist, elbow, or shoulder (Pagano, Fitzpatrick, & Turvey, 1993). In addition, Hajnal, Fonseca, Harrison, Kinsella-Shaw, and Carello (2007) found that perceivers are equally sensitive and accurate when perceiving length with the foot as when they perceive this property with the hand (see also Stephen & Hajnal, 2011).

The third reason for using dynamic touch was that a functional organization of calibration has already been demonstrated in this paradigm (Stephen & Hajnal, 2011; Withagen & Michaels, 2004). In the study by Withagen and Michaels (2004), participants were to perceive the length of wooden rods with both their right hand and their left hand. In the feedback phase, participants received feedback on their length estimates with their right hand. It was found that participants calibrated to the length of wooden rods and a transfer from the right to the left hand was observed. Recently, Stephen and Hajnal (2011) demonstrated that calibration of length perception by dynamic touch also transfers between the hand and the foot. In the present experiment, we tested whether *attunement* also follows this functional organization. Does learning to detect a specifying variable with one limb transfer to the all other limbs?

## Experiment

In the present study, we aimed to test for transfer of attunement in length perception by dynamic touch. To this end, a pretest-feedback-posttest design was used. In the test blocks, participants were to judge the length of rods with their feet as well as with their hands. In the feedback blocks, participants were to judge the length with only one of their limbs. After each judgment in the feedback blocks, participants received visual information about the actual length of the wielded rod. To find out whether attunement occurred, we tested for each individual participant which mechanical variables constrained the length judgments of each limb in each test phase. If attunement follows a functional organization, learning to detect a specifying variable with one effector should transfer to all the other effectors. If, on the other hand, attunement is anatomically specific, it should be confined to the trained limb and no transfer should occur.

### Participants

Sixty participants (28 men and 32 women) between age 18 and 57 years (mean age 22.5, standard deviation [SD] = 5.10) volunteered to participate and gave their informed consent. Fifty-seven of them were right-handed, and three were left-handed. The experiment was approved by the local institution’s ethics committee.

### Apparatus and materials

To prevent the participants from identifying individual rods, two distinct sets of rods were used: one set was used in the test phases, and the other set was used in the feedback phases. Each set consisted of thirteen rods made of carbon pipes and solid wood, steel, and aluminium. Within each set, the rods also differed in length and diameter. Each rod was affixed to an 11.5-cm plastic handle, preventing the participants from feeling the material and diameter of the wielded rods. The geometrical and mechanical properties of the rods are provided in the Appendix.

The rods in the two sets were chosen so that for each set *I*_*1*_ and *M* had low correlations with length (Table 1). The reason for this was twofold. First, when actual length and the nonspecifying variables *I*_*1*_ and *M* correlate weakly with each other, it is easier to reveal whether participants detect a specifying or a nonspecifying variable. Second, the low correlations between actual length and *I*_*1*_ and *M*, imply that they are poor variables to use for length judgments and therefore will yield clearly detectable errors in the feedback. This increases the chance that a change in variable usage will occur (Jacobs, Runeson, & Michaels, 2001; Michaels et al., 2008; Rop & Withagen, 2014).

**Table 1:** Correlations between the logarithms of the candidate variables and actual length

	Length	*I*_*1*_	*M*
Test rod set			
Length	-	0.183	0.137
*I*_*1*_	-	-	0.949
*M*	-	-	-
Feedback rod set			
Length	-	0.082	0.203
*I*_*1*_	-	-	0.959
*M*	-	-	-

We decided to examine length perception by dynamic touch in a horizontal wielding condition. In their study on transfer of calibration between hand and foot, Stephen and Hajnal (2011) used a vertical wielding condition (see also Hajnal et al., 2007). However, the horizontal wielding condition is more frequently used, especially in studies on attunement in length perception by dynamic touch (Arzamarski et al., 2010; Menger & Withagen, 2009; Michaels et al., 2008; Wagman et al., 2001; Withagen & Michaels, 2005). Consequently, the mechanical variables that are involved in learning to perceive length by dynamic touch are better understood in the horizontal wielding condition than in the vertical wielding condition. Hence, we opted for the former and not for the latter.

**Fig. 1:**
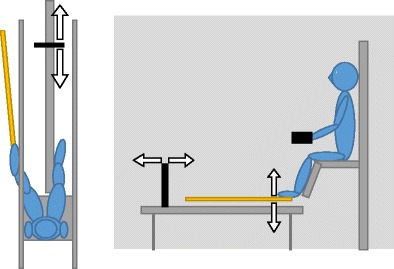
Experimental setup as seen from above (left) and from the side (right)

We used a slightly altered version of the apparatus that is commonly used in dynamic touch studies. The participants sat on a chair that was mounted on a platform of a hoist system (Fig. 1). This system allowed for winching the platform, needed to provide participants with enough space for unrestricted wielding of the rod around the ankle. Attached to the chair were two leg supports against which the participants rested their legs. Their legs were loosely fastened to these rests by Velcro, ensuring that their legs did not change position during the experiment. In front of the chair, there was a rail with a small planar surface attached to it. Participants could move this planar surface along the rail using a remote control. On each side of the rail, there was an armrest in which the forearm was positioned during the length judgments with the hands. Between the rail and each armrest was a curtain that blocked the participant’s vision such that the wielded rod was not visible. The participants put their hand or feet through one of the openings in the curtain to make the length judgments.

To judge the length with their feet, participants had to wear a one-of-a-kind sandal. To a standard sandal (Mountain Peak, size 8.5, USA size), we bolted metal sheets with a holder in which the rods could be tightly secured (total weight 0.81 kg). During the length judgments with the feet, the rods were placed in the holder such that the end of the handle coincided with the tip of the toes of the participant.

### Design and procedure

The experiment was conducted over the course of two consecutive days. We created 4 groups of 15 participants: 1 group received feedback on the length judgments with the right hand; 1 group on the judgments with the left hand; 1 group on the judgments with the right foot; and 1 group on the judgments with the left foot. Participants were randomly assigned to one of the groups. The first day consisted of the pretest and two feedback blocks; the second day consisted of two feedback blocks and the posttest. In both the pretest and the posttest, participants were to estimate the length of the rods with each of their limbs. They had to position the planar surface at the maximum distance reachable with the rod, that is, such that it coincided with the distal end of the rod. With each limb, participants had to judge the lengths of the 13 rods of the test set twice and the order of presentation was randomized. For the judgments with the hands, the chair was brought to regular sitting height, such that the hands were at the same height as the rail with the attached planar surface. For the judgements with the feet, the chair was lifted to a height such that the feet were at the height of this rail. To circumvent any order effects, the sequence of the nontrained limbs was randomized for each participant in both test phases. However, the trained limb was tested last. This was done to prevent the participants in the posttest from discovering the lengths of the rods in the test set with their trained limb and then using this information in the length estimates with the other limbs.

In the feedback blocks, participants were to perceive the length of the rods with one of their limbs. As mentioned earlier, participants were randomly assigned to one of the four groups, each group training one of the limbs. The feedback blocks consisted of 26 trials—each rod of the feedback rod set was presented twice in a randomized order. After the participant had positioned the planar surface at the perceived distance reachable with the rod, she was to touch the curtain with the rod. This led to a curtain displacement that provided both visual (Withagen & van Wermeskerken, 2009) and haptic feedback (Stephen & Arzamarski, 2008) on the length of the rod. When participants were unable to properly see where the rod touched the curtain, the experimenter touched the curtain at the location of the distal end of the rod.

Because a change in information usage has been found to be accompanied by a change in the wielding behaviour (Riley, Wagman, Santana, Carello, & Turvey, 2002; Michaels & Isenhower, 2011; Menger & Withagen, 2009), the participants could freely wield the rods, with the exception that they were not to touch the floor or the curtain during the length estimates. Moreover, to prevent the participants from comparing the length of the subsequent rods, they were to position the surface at the proximal end of the rail after each trial in both the test phases and the feedback phases.

## Results

**Fig. 2:**
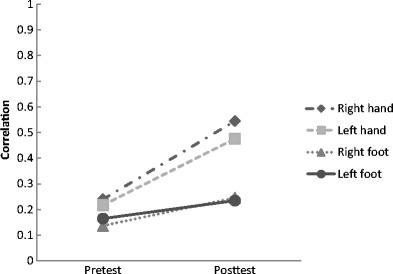
The (absolute) correlations of perceived length and actual length for each limb and each test phase, collapsed over training groups

We first tested whether the participants’ length estimates were more closely tied to actual length after feedback. For each individual and each limb, we computed the Pearson product-moment correlation of perceived length and actual length in both the pretest and the posttest. We conducted repeated-measures ANOVA on the absolute correlations^1^ with test (pretest, posttest) and limb (right hand, left hand, right foot, left foot) as within factors and group (right hand, left hand, right foot, left foot) as between factor. When the assumption of sphericity was violated (*p* < 0.05), we used the Huynh-Feldt correction. The main effect of test, *F*(1, 56) = 100.40, *p* < 0.001, indicates that the correlation of perceived length and actual length was significantly higher in the posttest (0.384) than in the pretest (0.190). We also found a main effect of limb, *F*(2.85, 159.74) = 50.88, *p* < 0.001. Pairwise comparisons, with Bonferroni correction, revealed that the left foot (0.200) and the right foot (0.193) did not differ from each other, nor did the right hand (0.404) differ from the left hand (0.353) (*p*s > 0.05). However, both the right hand and the left hand differed from both feet (*p*s < 0.05). We found no main effect of group or significant interactions of group x limb and group x test (*p*s > 0.05). The ANOVA revealed a test x limb interaction, *F*(3, 168) = 19.54, *p* < 0.001. As shown in Fig. 2, the hands improved more than the feet.

Moreover, we found a test x limb x group interaction, *F*(9, 168) = 2.89, *p* < 0.01, indicating that the change in correlation of perceived length and actual length over the course of the experiment not only differed for the different limbs but also depended on which limb was trained.

**Fig. 3:**
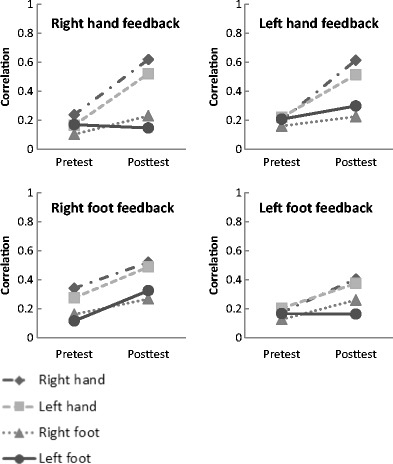
The (absolute) correlations of perceived length and actual length for each limb and each test phase broken down by feedback group

To reveal the origin of the above interaction effect, we conducted repeated-measures ANOVAs with test (pretest, posttest) and limb (right hand, left hand, right foot, left foot) as within factors for each group separately. Figure 3 depicts the correlations of perceived length and actual length for each limb and each test phase, broken down by group. For the group of participants who trained to perceive length with their right hand, we found a main effect of test, *F*(1, 14) = 71.62, *p* < 0.001, and a main effect of limb, *F*(3, 42) = 21.36, *p* < 0.001. Pairwise comparisons, with Bonferroni correction, revealed that the right hand and the left hand differed significantly from both the right foot and the left foot (*p*s < 0.001). We also found an interaction effect of test x limb, *F*(3, 42) = 13.37, *p* < 0.001. As displayed in Fig. 3, the improvement in the length estimates with the right hand transferred almost perfectly to the left hand, but not to the feet.

Similar results were found for the group who trained the left hand. We found a main effect of test, *F*(1, 14) = 27.24, *p* < 0.001, and a main effect of limb, *F*(3, 42) = 10.85, *p* < 0.001. The hands did not differ from each other, nor did the feet (*p*s > 0.05). In contrast, the left hand differed from the left foot, and the right hand differed from both the left foot and the right foot (*p*s < 0.05). More importantly, we found an interaction of test x limb, *F*(3, 42) = 8.64, *p* < 0.001. The improvement that was apparent in the trained left hand transferred to the right hand but only led to a minor improvement in the feet (Fig. 3).

When training the right foot, the transfer effects were different. Again, we found a main effect of test, *F*(1, 14) = 18.98, *p* < 0.01, and a main effect of limb, *F*(3, 42) = 13.76, *p* < 0.001. Pairwise comparisons revealed that the right hand and left hand differed from the right foot and the left foot (*ps* < 0.05). However, we found no significant interaction of test x limb, *F*(3, 42) = 0.99, *p* > 0.05, indicating that the improvement in the length judgments with the right foot transferred to all the other limbs (Fig. 3).

Also in the group who trained the left foot, we found a main effect of test, *F*(1, 14) = 11.47, *p* < 0.01, and a main effect of limb, *F*(3, 42) = 7.42, *p* < 0.001. As in the other groups, the hands did not differ significantly from each other, nor did the feet (*p*s > 0.05). In contrast, the right hand differed from the left foot (*p* < 0.05). We also found an interaction effect of test x limb, *F*(2.09, 29.25) = 3.50, *p* < 0.05). Surprisingly, after training the left foot, this foot did not show improvement in the length judgments, but the other limbs did (Fig. 3). All in all, we found that in training length perception with one of the feet, the other limbs improve; but in training one of the hands, the transfer is confined to the other hand.

Although the above analyses gave insight into improvements in length perception and transfer thereof, they provide no insight into the process of attunement, that is, the changes in what information is exploited over the course of the experiment. Below we will look into that for each of the groups separately.

### Attunement in the different training groups

#### Training the right hand

We first tested which mechanical variables participants exploited in the test phases. For each participant and each limb, we computed the correlations of perceived length and actual length and the nonspecifying variables *I*_*1*_ and *M*. In computing these correlations, we used the logarithms of these variables, because *I*_*1*_ increases with the cube of length (Withagen & Michaels, 2005; Withagen & van Wermeskerken, 2009). The correlations averaged over participants are displayed in Table 2. To test whether participants relied on a specifying or a nonspecifying variable, we compared the correlation of perceived length and actual length with the correlation of perceived length and most highly correlated nonspecifying variable (*I*_*1*_ or *M*).

**Table 2:** The (absolute) correlations of perceived length and actual length (L), M and I_1_ in the pretest and posttest for participants who trained the right hand

Limb	Pretest			Posttest		
	L	*I*_*1*_	*M*	L	*I*_*1*_	*M*
Left hand	0.153	0.819	0.810	0.515	0.286	0.255
Right hand	0.216	0.843	0.827	0.618	0.358	0.283
Left foot	0.165	0.725	0.779	0.145	0.464	0.487
Right foot	0.098	0.738	0.775	0.242	0.472	0.494

In the pretest, participants relied on a nonspecifying variable with each limb—the correlation with *I*_*1*_ was significantly higher than the correlation with actual length for both the right hand, *t*(14) = 9.77, *p* < 0.001, and the left hand, *t*(14) = 12.17, *p* < 0.001; and the correlation with *M* was significantly higher than the correlation with actual length for both the right foot, *t*(14) = 12.81, *p* < 0.001, and the left foot, *t*(14) = 11.04, *p* < 0.001. Hence, in line with earlier studies (Hajnal et al., 2007; Stephen & Hajnal, 2011), we found that participants are capable of detecting nonspecifying variables with their feet. After training the right hand, participants attuned to the specifying variable with both hands—the correlation with actual length was significantly higher than the correlation with *I*_*1*_ for the right hand, *t*(14) =3.40, *p* < 0.001, and the left hand, *t*(14) = 3.84, *p* < 0.001. However, participants continued to rely on a nonspecifying variable with their feet—the correlation with *M* was significantly higher than the correlation with actual length for the right foot, *t*(14) = 2.61, *p* < 0.05, and the left foot, *t*(14) = 4.34, *p* < 0.001. Hence, when the right hand was trained, participants attuned to the specifying variable with both hands but maintained reliance on the variable that was detected in the pretest with their feet.

Because individuals have been found to vary in their perceptual learning capacities (Menger & Withagen, 2009; Rop & Withagen, 2014; Withagen & Caljouw, 2011; Withagen & van Wermeskerken, 2009), we analyzed the learning trajectories of each individual participant as well. To test whether an individual relied on a specifying variable or a nonspecifying variable, we compared for each participant in each test phase the correlation of perceived length and actual length with the correlation of perceived length and the most highly correlated nonspecifying variable, using Williams’ t-statistic (May & Hittner, 1997; Williams, 1959). Only when the difference between the correlations was significant, we concluded that the participant detected a specifying or a nonspecifying variable (depending on which correlation was the highest).

An overview of the results is given in Table 3. In the pretest, all but five participants relied on nonspecifying variables with all of their limbs. When provided with feedback on the right hand, the perceptual performances of the individuals diverged. Although with the right hand none of the participants continued to rely on the variable, they detected in the pretest only five participants (Participants 1, 4, 5, 11, and 12) learned to detect a specifying variable with this hand. This is in line with earlier studies on length perception with the right hand that showed that participants vary in their ability to learn to detect specifying variables (Menger & Withagen, 2009; Rop & Withagen, 2014; Withagen & van Wermeskerken, 2009). Moreover, the present study demonstrated that participants also vary in the type of transfer. Of the five participants who learned to detect the specifying variable with their right hand, only one (Participant 11) succeeded in doing so with the left hand. The other participant (Participant 10) who detected a specifying variable with the left hand in the posttest did not do so with the trained right hand. Although the analyses at the group level demonstrated that participants maintained reliance on a nonspecifying variable with their feet after feedback, only three participants (Participants 7, 8, and 11) did so with both feet, and two participants (Participants 4 and 13) did so with either the right foot or the left foot. The other participants demonstrated changes in variable usage with their feet after having been provided with feedback on the right hand. This suggests that attunement is not confined to the limb that is trained, but the type of transfer depends on the individual.

**Table 3:** The (absolute) correlations of perceived length and actual length (L), and perceived length and the most highly correlating nonspecifying variable for both test phases for each individual participant who received feedback for the right hand

Pp	Pretest				Posttest			
	Right hand	Left hand	Right foot	Left foot	Right hand	Left hand	Right foot	Left foot
1	*I*_1_* 0.922	M* 0.913	M* 0.715	M* 0.829	*I*_1_ 0.448	*I*_1_ 0.142	*I*_1_ 0.437	*I*_1_ 0.397
	*L* 0.172	*L* 0.130	*L*0.107	*L* 0.241	L* 0.815	*L* 0.530	*L* 0.457	*L* 0.173
2	*I*_1_* 0.712	M* 0.872	*I*_1_* 0.646	M* 0.697	*M* 0.436	*M* 0.248	*M* 0.418	*M* 0.221
	*L* 0.270	*L* 0.010	*L* 0.043	*L* 0.124	*L* 0.617	*L* 0.558	*L* 0.291	*L* 0.140
3	*I*_1_* 0.870	M*0.771	M*0.833	M* 0.606	*I*_1_ 0.337	*I*_1_ 0.267	*M* 0.290	*I*_1_ 0.273
	*L* 0.114	*L* 0.122	*L* 0.224	*L* 0.095	*L* 0.701	*L* 0.307	*L* 0.072	*L* 0.132
4	*I*_1_* 0.735	*I*_1_ 0.557	*I*_1_* 0.690	M* 0.789	*I*_1_ 0.135	*M* 0.189	M* 0.692	*I*_1_ 0.500
	*L* 0.141	*L* 0.285	*L* 0.033	*L* 0.057	L* 0.753	*L* 0.279	*L* 0.272	*L* 0.221
5	M* 0.896	M* 0.859	*I*_1_* 0.554	M* 0.729	*M* 0.174	*I*_1_ 0.125	*M* 0.557	*M* 0.314
	*L* 0.158	*L* 0.023	*L* 0.067	*L* 0.214	L* 0.714	*L* 0.496	*L* 0.095	*L* 0.043
6	M* 0.973	M* 0.709	M* 0.859	*M* 0.432	*I*_1_ 0.239	*M* 0.234	*I*_1_ 0.366	*M* 0.190
	*L* 0.088	*L* 0.001	*L* 0.133	*L* 0.255	*L* 0.475	*L* 0.527	*L* 0.332	*L* 0.031
7	*I*_1_ 0.489	*I*_1_* 0.898	M* 0.686	M* 0.879	*I*_1_ 0.573	*I*_1_ 0.551	M* 0.765	M* 0.822
	*L* 0.195	*L* 0.168	*L* 0.061	*L* 0.089	*L* 0.716	*L* 0.765	*L* 0.118	*L* 0.199
8	*I*_1_* 0.886	*I*_1_* 0.772	M* 0.721	M* 0.823	*M* 0.759	*M* 0.612	M* 0.768	*I*_1_* 0.605
	*L* 0.209	*L* 0.266	*L* 0.065	*L* 0.214	*L* 0.496	*L* 0.569	*L* 0.388	*L* 0.041
9	*I*_1_* 0.892	*I*_1_* 0.908	M* 0.893	M* 0.812	*I*_1_ 0.337	*I*_1_ 0.354	*M* 0.147	*M* 0.501
	*L* 0.352	*L* 0.149	*L* 0.097	*L* 0.280	*L* 0.480	*L* 0.345	*L* 0.111	*L* 0.521
10	*I*_1_* 0.907	*I*_1_* 0.869	*I*_1_* 0.915	*I*_1_* 0.885	*M* 0.161	*I*_1_ 0.112	*M* 0.416	*M* 0.514
	*L* 0.250	*L* 0.095	*L* 0.028	*L* 0.058	*L* 0.204	L* 0.673	*L* 0.400	*L* 0.052
11	*I*_1_ 0.771	*I*_1_* 0.845	M* 0.725	M* 0.844	*I*_1_ 0.349	*I*_1_ 0.426	*I*_1_* 0.878	*I*_1_* 0.815
	*L* 0.551	*L* 0.188	*L* 0.046	*L* 0.015	L* 0.809	L* 0.781	*L* 0.052	*L* 0.020
12	*I*_1_* 0.751	*I*_1_* 0.760	*M* 0.470	M* 0.657	*I*_1_ 0.229	*M* 0.238	*I*_1_ 0.104	*M* 0.201
	*L* 0.295	*L* 0.384	*L* 0.016	*L* 0.169	L* 0.807	*L* 0.189	*L* 0.426	*L* 0.133
13	M* 0.917	M* 0.930	M* 0.863	M* 0.887	*I*_1_ 0.547	*I*_1_ 0.540	*I*_1_ 0.433	M* 0.701
	*L* 0.129	*L* 0.091	*L* 0.216	*L* 0.188	*L* 0.438	*L* 0.610	*L* 0.263	*L* 0.132
14	M* 0.786	*I*_1_* 0.734	M* 0.785	M* 0.709	*I*_1_ 0.610	*I*_1_ 0.598	*I*_1_ 0.548	*I*_1_ 0.430
	*L* 0.174	*L* 0.111	*L* 0.121	*L* 0.281	*L* 0.555	*L* 0.367	*L* 0.097	*L* 0.146
15	M* 0.955	M* 0.852	M* 0.882	M* 0.806	*M* 0.448	*M* 0.286	*M* 0.381	*M* 0.520
	*L* 0.069	*L* 0.245	*L* 0.198	*L* 0.178	*L* 0.188	*L* 0.395	*L* 0.181	*L* 0.130

Asterisk indicates a significant difference (*p* < 0.05, two-tailed) between the correlation with actual length and the correlation with the most highly correlating nonspecifying variable.

#### Training the left hand

In the group of participants who trained the left hand, we also found that participants relied on nonspecifying variables in the pretest—the correlation with *I*_*1*_ was significantly higher than the correlation with actual length for both the right hand, *t*(14) = 7.21, *p* < 0.001, and the left hand, *t*(14) = 5.98, *p* < 0.001; and the correlation with *M* was significantly higher than the correlation with actual length for both the right foot, *t*(14) = 7.06, *p* < 0.001, and the left foot, *t*(14) = 6.40, *p* < 0.001 (Table 4). After feedback, participants no longer relied on these variables, but they did not succeed in detecting a specifying variable. For none of the limbs, we found a significant difference between the correlation with actual length and the correlation with the most highly correlated nonspecifying variable (*p*s > 0.05). This may indicate that participants switched between mechanical variables in the posttest or that they relied on an informational variable that we did not consider (Withagen & van Wermeskerken, 2009).

**Table 4:** The (absolute) correlations of perceived length and actual length (L), M and I_1_ in the pretest and posttest for participants who trained the left hand

Limb	Pretest			Posttest		
	L	*I*_*1*_	*M*	L	*I*_*1*_	*M*
Left hand	0.205	0.787	0.772	0.511	0.456	0.369
Right hand	0.207	0.786	0.743	0.602	0.439	0.374
Left foot	0.189	0.719	0.741	0.291	0.325	0.318
Right foot	0.157	0.679	0.708	0.218	0.361	0.377

Our analyses of the individuals revealed that all but two participants (Participants 2 and 7) tended to rely on nonspecifying variables in the pretest (Table 5). After receiving feedback on the left hand, only Participant 10 still relied on a nonspecifying variable with this limb. Three participants (Participants 6, 12, and 14) learned to rely on a specifying variable with the trained left hand. Of these three, Participant 14 also attuned to a specifying variable with the right hand and Participant 6 even attuned to a specifying variable with the right hand as well as with the left foot. In contrast, Participant 12 did not show transfer of attunement. In fact, this participant kept relying on a nonspecifying variable with the right foot. Although they did not attune to a specifying variable with the left hand, three participants (Participants 2, 3, and 11) attuned to a specifying variable with at least one of the other limbs.

**Table 5:** The (absolute) correlations of perceived length and actual length (L), and perceived length and the most highly correlating nonspecifying variable for both test phases for each individual participant who received feedback for the left hand

Pp	Pretest				Posttest			
	Right hand	Left hand	Right foot	Left foot	Right hand	Left hand	Right foot	Left foot
1	M* 0.938	*I*_1_* 0.847	M* 0.887	M* 0.866	*I*_1_ 0.475	*I*_1_ 0.401	*M* 0.295	*M* 0.461
	*L* 0.080	*L* 0.046	*L* 0.090	*L* 0.396	*L* 0.607	*L* 0.194	*L* 0.255	*L* 0.116
2	*I*_1_ 0.561	*I*_1_ 0.501	*I* 0.442	*I* 0.424	*I*_1_ 0.244	*I*_1_ 0.328	*I*_1_ 0.144	*I*_1_ 0.056
	*L* 0.343	*L* 0.151	*L* 0.444	*L* 0.147	*L* 0.532	*L* 0.376	L* 0.593	*L* 0.168
3	*I*_1_* 0.767	*I*_1_* 0.948	M* 0.822	M* 0.850	*M* 0.385	*M* 0.415	*M* 0.457	*M* 0.220
	*L* 0.286	*L* 0.197	*L* 0.155	*L* 0.060	L*0.765	*L* 0.602	*L* 0.340	L* 0.679
4	M* 0.815	M* 0.771	*I*_1_* 0.774	*I*_1_* 0.652	*M* 0.463	*M* 0.593	*M* 0.603	*M* 0.099
	*L* 0.020	*L* 0.173	*L* 0.127	*L* 0.045	*L* 0.602	*L* 0.162	*L* 0.180	*L* 0.155
5	*I*_1_* 0.688	*I*_1_* 0.736	M* 0.586	*M* 0.388	*I*_1_ 0.436	*I*_1_ 0.542	*M* 0.016	*I*_1_ 0.665
	*L* 0.120	*L* 0.296	*L* 0.126	*L* 0.111	*L* 0.486	*L* 0.38	*L* 0.061	*L* 0.283
6	*I*_1_* 0.849	*I*_1_ 0.333	M* 0.806	*I*_1_* 0.753	*I*_1_ 0.440	*I*_1_ 0.266	M* 0.511	*I*_1_ 0.280
	*L* 0.342	*L* 0.640	*L* 0.105	*L* 0.176	L* 0.848	L* 0.722	*L* 0.003	L* 0.843
7	*M* 0.236	*I*_1_ 0.585	*I*_1_ 0.258	*I*_1_ 0.291	*M* 0.429	*I*_1_ 0.278	*M* 0.237	*M* 0.144
	*L* 0.275	*L* 0.216	*L* 0.053	*L* 0.290	*L* 0.752	*L* 0.508	*L* 0.333	*L* 0.398
8	*I*_1_* 0.867	M* 0.844	M* 0.812	M* 0.783	*I*_1_* 0.815	*I*_1_ 0.589	*I*_1_ 0.494	M* 0.590
	*L* 0.131	*L* 0.028	*L* 0.182	*L* 0.149	*L* 0.464	*L* 0.617	*L* 0.093	*L* 0.115
9	M* 0.857	M* 0.907	M* 0.833	M* 0.870	*I*_1_ 0.648	*I*_1_ 0.628	*I*_1_ 0.246	*I*_1_ 0.018
	*L* 0.007	*L* 0.100	*L* 0.093	*L* 0.120	*L* 0.450	*L* 0.381	*L* 0.031	*L* 0.011
10	*I*_1_* 0.907	M* 0.821	M* 0.779	M* 0.904	M* 0.787	*I*_1_* 0.832	M* 0.631	M* 0.649
	*L* 0.034	*L* 0.092	*L* 0.098	*L* 0.107	*L* 0.015	*L* 0.095	*L* 0.169	*L* 0.003
11	*I*_1_* 0.816	*I*_1_* 0.934	*M* 0.645	M* 0.791	*M* 0.263	*M* 0.396	*I*_1_ 0.074	*I*_1_ 0.088
	*L* 0.512	*L* 0.131	*L* 0.241	*L* 0.187	L* 0.769	*L* 0.720	*L* 0.231	*L* 0.374
12	M* 0.793	M* 0.852	*M* 0.466	*I*_1_* 0.861	*I*_1_ 0.252	*I*_1_ 0.422	*I*_1_* 0.708	*I*_1_ 0.464
	*L* 0.088	*L* 0.180	*L* 0.114	*L* 0.161	*L* 0.597	L* 0.782	*L* 0.186	*L* 0.094
13	*I*_1_ 0.577	*I*_1_ 0.443	M* 0.573	*I*_1_* 0.662	*I*_1_ 0.414	*I*_1_ 0.558	*M* 0.558	*M* 0.505
	*L* 0.185	*L* 0.368	*L* 0.039	*L* 0.231	*L* 0.291	*L* 0.494	*L* 0.106	*L* 0.071
14	M* 0.902	M* 0.931	M* 0.860	M* 0.927	*I*_1_ 0.328	*I*_1_ 0.251	*M* 0.323	*M* 0.086
	*L* 0.055	*L* 0.105	*L* 0.188	*L* 0.191	L*0.835	L* 0.781	*L* 0.292	*L* 0.226
15	*I*_1_* 0.809	*I*_1_* 0.863	*I*_1_* 0.746	*I*_1_ 0.711	*I*_1_ 0.626	*I*_1_ 0.483	*I*_1_ 0.417	*I*_1_ 0.703
	*L* 0.512	*L* 0.227	*L* 0.259	*L* 0.42	*L* 0.442	*L* 0.362	*L* 0.298	*L* 0.349

Asterisk indicates a significant difference (*p* < 0.05, two-tailed) between the correlation with actual length and the correlation with the most highly correlating nonspecifying variable.

#### Training the right foot

The participants who trained the right foot also generally relied on nonspecifying variables in the pretest—their length judgments correlated more highly with the nonspecifying variable *I*_*1*_ than with a specifying variable for the right hand, *t*(14) = 4,59, *p* < 0.001, the left hand, *t(14)* = 5.50, *p* < 0.001, the right foot, *t*(14) = 8.84, *p* < 0.001, and the left foot *t*(14) = 7.05, *p* < 0.001 (Table 6). After the participants were provided with feedback on their right foot, they did not continue to rely on the nonspecifying variables. However, they also did not succeed in learning to detect a specifying variable with one of their limbs. We did not find a significant difference between the correlation with actual length and the correlation with the most highly correlated nonspecifying variable for any limb (*p*s > 0.05).

**Table 6:** The (absolute) correlations of perceived length and actual length (L), M and I_1_ in the pretest and posttest for participants who trained the right foot

Limb	Pretest			Posttest		
	L	*I*_*1*_	*M*	L	*I*_*1*_	*M*
Left hand	0.265	0.815	0.766	0.470	0.521	0.404
Right hand	0.336	0.799	0.730	0.491	0.526	0.410
Left foot	0.107	0.747	0.734	0.318	0.405	0.387
Right foot	0.145	0.768	0.731	0.264	0.391	0.407

As in the other groups, the majority of participants generally relied on nonspecifying variables in the pretest (Table 7). Surprisingly, Participant 8 detected a specifying variable with the left hand before receiving feedback. After training the right foot, all but two participants (Participants 12 and 14) demonstrated changes in variable usage with at least one of their limbs. However, only Participant 2 detected a specifying variable with this foot but not with the other limbs. Participant 15, on the other hand, relied on a specifying variable in the posttest with both hands, Participant 9 did so with the right hand, and Participants 13 and 3 did so with their left hands. Interestingly, although Participant 13 learned to detect a specifying variable with the left hand, this participant still relied on a nonspecifying variable in the posttest with the trained right foot. Apparently, training the right foot can result in attunement to specifying variables with the other limbs, even if the participant maintains reliance on a nonspecifying variable with the right foot.

**Table 7:** The (absolute) correlations of perceived length and actual length (L) and perceived length and the most highly correlating nonspecifying variable for both test phases for each individual participant who received feedback for the right foot

Pp	Pretest				Posttest			
	Right hand	Left hand	Right foot	Left foot	Right hand	Left hand	Right foot	Left foot
1	M* 0.928	M* 0.954	*I*_1_* 0.797	M* 0.829	*I*_1_ 0.261	*I*_1_ 0.146	*M*_1_ 0.326	*M*_1_ 0.220
	*L* 0.106	*L* 0.010	*L* 0.121	*L* 0.013	*L* 0.295	*L* 0.261	*L* 0.463	*L* 0.357
2	*I*_1_ 0.568	*I*_1_ 0.579	*I*_1_* 0.522	*I*_1_ 0.522	*I*_1_ 0.383	*I*_1_ 0.702	*I*_1_ 0.255	*M* 0.291
	*L* 0.463	*L* 0.233	*L* 0.011	*L* 0.28	*L* 0.692	*L* 0.618	L* 0.681	*L* 0.566
3	*I*_1_ 0.705	*I*_1_* 0.760	*I*_1_ 0.497	*I*_1_* 0.694	*I*_1_ 0.373	*I*_1_ 0.371	*I*_1_ 0.373	*I*_1_ 0.286
	*L* 0.590	*L* 0.441	*L* 0.351	*L* 0.185	*L* 0.531	L* 0.775	*L* 0.223	*L* 0.590
4	*I*_1_* 0.788	*I*_1_* 0.715	*I*_1_* 0.727	*I*_1_* 0.816	*I*_1_ 0.254	*I*_1_ 0.657	*M* 0.383	*I*_1_ 0.472
	*L* 0.467	*L* 0.268	*L* 0.180	*L* 0.075	*L* 0.647	*L* 0.488	*L* 0.432	*L* 0.494
5	*I*_1_ 0.499	*I*_1_ 0.571	*I*_1_* 0.796	*M* 0.154	*I*_1_* 0.866	*I*_1_* 0.804	*I*_1_ 0.543	*I*_1_* 0.801
	*L* 0.674	*L* 0.378	*L* 0.238	*L* 0.150	*L* 0.239	*L* 0.371	*L* 0.127	*L* 0.242
6	*I*_1_* 0.902	*I*_1_* 0.876	*I*_1_* 0.939	M* 0.888	*I*_1_ 0.691	*I*_1_ 0.693	M* 0.658	*M* 0.216
	*L* 0.313	*L* 0.300	*L* 0.056	*L* 0.012	*L* 0.479	*L* 0.547	*L* 0.063	*L* 0.222
7	*I*_1_* 0.870	*I*_1_* 0.897	*I*_1_ *0.839	*I*_1_ *0.847	*I*_1_ 0.601	M* 0.661	*M* 0.323	*I*_1_* 0.577
	*L* 0.058	*L* 0.057	*L* 0.156	*L* 0.162	*L* 0.217	*L* 0.072	*L* 0.038	*L* 0.088
8	*I*_1_ 0.298	*I*_1_ 0.354	*I*_1_ 0.561	*I*_1_ 0.496	*I*_1_ 0.296	*I*_1_ 0.469	*I*_1_ 0.452	*I*_1_ 0.577
	*L* 0.516	L* 0.749	*L* 0.302	*L* 0.211	*L* 0.660	*L* 0.648	*L* 0.324	*L* 0.374
9	*I*_1_* 0.683	*I*_1_* 0.855	M* 0.719	*I*_1_ 0.366	*I*_1_ 0.358	*I*_1_ 0.159	*M* 0.097	*I*_1_ 0.162
	*L* 0.145	*L* 0.084	*L* 0.023	*L* 0.102	L* 0.724	*L* 0.325	*L* 0.078	*L* 0.412
10	I* 0.834	M* 0.832	M* 0.803	M* 0.715	*I*_1_* 0.716	*I*_1_ 0.426	*M* 0.477	*I*_1_ 0.276
	*L* 0.232	*L* 0.206	*L* 0.149	*L* 0.007	*L* 0.206	*L* 0.217	*L* 0.122	*L* 0.057
11	*I*_1_* 0.934	*I*_1_* 0.920	*I*_1_* 0.865	*I*_1_* 0.845	*I*_1_ 0.294	*I*_1_ 0.054	*I*_1_ 0.117	*M* 0.362
	*L* 0.151	*L* 0.115	*L* 0.026	*L* 0.082	*L* 0.232	*L* 0.247	*L* 0.331	*L* 0.095
12	M* 0.964	M* 0.930	M* 0.867	M* 0.954	*I*_1_* 0.843	*I*_1_* 0.791	M* 0.693	*I*_1_* 0.675
	*L* 0.009	*L* 0.007	*L* 0.156	*L* 0.093	*L* 0.433	*L* 0.426	*L* 0.074	*L* 0.042
13	*I*_1_ 0.778	*I*_1_* 0.856	*I*_1_* 0.797	*I*_1_* 0.924	*M* 0.306	*I*_1_ 0.132	M* 0.755	*M* 0.674
	*L* 0.493	*L* 0.428	*L* 0.065	*L* 0.164	*L* 0.607	L* 0.722	*L* 0.184	*L* 0.303
14	*I*_1_* 0.825	M* 0.651	*I*_1_0.645	M* 0.775	*I*_1_* 0.834	*I*_1_* 0.876	M* 0.569	*M* 0.302
	*L* 0.246	*L* 0.039	*L* 0.255	*L* 0.002	*L* 0.105	*L* 0.261	*L* 0.081	*L* 0.369
15	*I*_1_* 0.741	*I*_1_* 0.878	M* 0.875	*I*_1_* 0.633	*I*_1_ 0.154	*M* 0.189	*M* 0.225	*I*_1_ 0.405
	*L* 0.335	*L* 0.399	*L* 0.055	*L* 0.060	L* 0.814	L* 0.671	*L* 0.526	*L* 0.403

Asterisk indicates a significant difference (*p* < 0.05, two-tailed) between the correlation with actual length and the correlation with the most highly correlating nonspecifying variable.

#### Training the left foot

At group level, we again found that participants relied on nonspecifying variables in the pretest (Table 8). The correlation with *I*_*1*_ was significantly higher than the correlation with actual length for the right hand, *t*(14) = 13.04, *p* < 0.001, and the left hand, *t*(14) = 9.46, *p* < 0.001; and the correlation with *M* was significantly higher than the correlation with actual length for the right foot, *t*(14) = 9.06, *p* < 0.001, and the left foot, *t*(14)= 8.54, *p* < 0.001. In the posttest, participants still relied on a nonspecifying variable with the left foot —the correlation with *M* was significantly higher than the correlation with actual length, *t*(14) = 3.28, *p* < 0.01. Hence, after having been provided feedback on the length perception with their left foot, participants continued to rely on a nonspecifying variable. For the other limbs, the correlation with actual length did not differ significantly from the correlation with the most highly correlated nonspecifying variable (*p*s > 0.05).

**Table 8:** The (absolute) correlations of perceived length and actual length (L), M and I_1_ in the pretest and posttest for participants who trained the left foot

Limb	Pretest			Posttest		
	L	*I*_*1*_	*M*	L	*I*_*1*_	*M*
Left hand	0.197	0.826	0.808	0.351	0.573	0.511
Right hand	0.160	0.841	0.806	0.390	0.590	0.507
Left foot	0.158	0.729	0.772	0.152	0.455	0.454
Right foot	0.128	0.764	0.796	0.250	0.323	0.310

All but four participants (Participants 1, 5, 8, and 13) detected nonspecifying variables in the pretest with all of their limbs (Table 9). After training the left foot, only 3 of 15 participants (Participants 3, 4, 13) still relied on a nonspecifying variable with this limb. Yet none of the participant succeeded in detecting a specifying variable with the left foot. However, for Participant 2, the training with the left foot resulted in an attunement to a specifying variable with both hands; and for Participant 11 the training yields such an attunement with the right hand. Hence, as for the right foot, training the left foot can result in an attunement with (one of) the other limbs.

**Table 9:** The (absolute) correlations of perceived length and actual length (L) and perceived length and the most highly correlating nonspecifying variable for both test phases for each individual participant who received feedback for the left foot

Pp	Pretest				Posttest			
	Right hand	Left hand	Right foot	Left foot	Right hand	Left hand	Right foot	Left foot
1	M* 0.570	M* 0.915	*M* 0.158	M* 0.910	*I*_1_ 0.164	*M* 0.671	*M* 0.639	*M* 0.246
	*L* 0.103	*L* 0.143	*L* 0.140	*L* 0.126	*L* 0.382	*L* 0.336	*L* 0.242	*L* 0.388
2	*I*_1_* 0.773	M* 0.913	*I*_1_* 0.884	*I*_1_* 0.712	*I*_1_ 0.364	*I*_1_ 0.428	*I*_1_ 0.651	*I*_1_ 0.422
	*L* 0.046	*L* 0.072	*L* 0.174	*L* 0.086	L* 0.758	L* 0.794	*L* 0.242	*L* 0.293
3	M* 0.945	*I*_1_* 0.896	M* 0.757	M* 0.877	*I*_1_* 0.806	*I*_1_* 0.798	*I*_1_ 0.363	*I*_1_* 0.673
	*L* 0.016	*L* 0.108	*L* 0.244	*L* 0.172	*L* 0.211	*L* 0.256	*L* 0.245	*L* 0.237
4	*I*_1_* 0.856	M*0.942	M* 0.904	M* 0.872	*I*_1_* 0.804	*I*_1_* 0.750	*M* 0.375	M* 0.906
	*L* 0.152	*L* 0.004	*L* 0.131	*L* 0.261	*L* 0.400	*L* 0.114	*L* 0.163	*L* 0.078
5	*I*_1_* 0.893	*M* 0.583	M* 0.900	M* 0.758	M* 0.726	*I*_1_ *0.780	*I*_1_ 0.426	*I*_1_ 0.345
	*L* 0.038	*L* 0.296	*L* 0.046	*L* 0.185	*L* 0.152	*L* 0.057	*L* 0.193	*L* 0.120
6	*I*_1_* 0.850	*I*_1_ *0.766	*M* *0.895	*I*_1_* 0.698	*I*_1_ 0.703	*I*_1_ 0.578	*I*_1_ 0.229	*I*_1_ 0.315
	*L* 0.151	*L* 0.143	*L* 0.124	*L* 0.174	*L* 0.461	*L* 0.480	*L* 0.031	*L* 0.237
7	M* 0.888	M* 0.925	M* 0.903	M* 0.867	M* 0.742	M* 0.740	*I*_1_ 0.446	*M* 0.339
	*L* 0.138	*L* 0.124	*L* 0.136	*L* 0.142	*L* 0.302	*L* 0.038	*L* 0.300	*L* 0.112
8	*I*_1_* 0.697	*I*_1_ 0.630	*M* 0.467	*I*_1_ 0.399	*I*_1_* 0.673	*I*_1_ 0.392	*M* 0.302	*M* 0.472
	*L* 0.215	*L* 0.444	*L* 0.047	*L* 0.330	*L* 0.084	*L* 0.023	*L* 0.382	*L* 0.155
9	*I*_1_* 0.866	*I*_1_* 0.792	M* 0.695	*I*_1_* 0.697	M* 0.653	*I*_1_ 0.479	*I*_1_ 0.362	*I*_1_ 0.469
	*L* 0.381	*L* 0.413	*L* 0.082	*L* 0.081	*L* 0.019	*L* 0.356	*L* 0.322	*L* 0.049
10	*I*_1_* 0.912	*I*_1_* 0.858	M* 0.832	M* 0.732	*I*_1_ 0.546	*I*_1_ 0.721	*I*_1_ 0.516	*I*_1_ 0.577
	*L* 0.221	*L* 0.258	*L* 0.229	*L* 0.347	*L* 0.457	*L* 0.399	*L* 0.389	*L* 0.145
11	*I*_1_* 0.937	*I*_1_* 0.820	*I*_1_* 0.906	M* 0.914	*I*_1_ 0.134	*M* 0.165	*M* 0.176	*M* 0.422
	*L* 0.224	*L* 0.291	*L* 0.028	*L* 0.154	L* 0.605	*L* 0.469	*L* 0.327	*L* 0.0453
12	*I*_1_* 0.806	*I*_1_* 0.712	M* 0.767	M* 0.744	*I*_1_ 0.621	*I*_1_ 0.342	*I*_1_ 0.126	*M* 0.038
	*L* 0.311	*L* 0.083	*L* 0.221	*L* 0.051	*L* 0.546	*L* 0.632	*L* 0.215	*L* 0.078
13	*I*_1_* 0.750	*I*_1_* 0.871	M* 0.840	*M* 0.406	*I*_1_* 0.867	*I*_1_ 0.558	*M* 0.361	*I*_1_* 0.651
	*L* 0.095	*L* 0.278	*L* 0.010	*L* 0.073	*L* 0.177	*L* 0.205	*L* 0.116	*L* 0.029
14	*I*_1_* 0.844	*I*_1_* 0.866	M* 0.737	M* 0.761	*I*_1_ 0.401	*I*_1_ 0.582	*M* 0.079	*I*_1_ 0.214
	*L* 0.094	*L* 0.057	*L* 0.131	*L* 0.083	*L* 0.508	*L* 0.267	*L* 0.199	*L* 0.202
15	*I*_1_* 0.798	*I*_1_* 0.782	*I*_1_* 0.726	M* 0.840	*I*_1_ 0.149	*I*_1_ 0.472	*I*_1_ 0.070	*M* 0.550
	*L* 0.181	*L* 0.190	*L* 0.173	*L* 0.078	*L* 0.489	*L* 0.484	*L* 0.351	*L* 0.088

Asterisk indicates a significant difference (*p* < 0.05, two-tailed) between the correlation with actual length and the correlation with the most highly correlating nonspecifying variable.

## Discussion

Earlier studies have tested whether transfer of calibration follows a functional organization—if one means to achieve a goal is calibrated are other means of achieving this goal calibrated as well, even when they are realized by different anatomical structures? Although functionally specific transfer of calibration has been observed (Rieser et al. 1995; Stephen & Hajnal, 2011; Withagen & Michaels, 2002, 2004), the evidence for such an organization is not unequivocal (Durgin et al., 2003; Bingham et al., 2014; Martin et al., 1996; Withagen & Michaels, 2007). In the present study, rather than calibration we considered the process of attunement and tested whether attunement follows a functional organization. Participants were trained to perceive length by dynamic touch with one of their limbs. We found that training one of the hands resulted in an improvement in length perception with both hands but not with the feet. Conversely, training one of the feet yielded an improvement in the perception of length of each limb. However, an examination of the mechanical variables that were exploited with the limbs revealed that participants only succeeded in detecting a specifying mechanical variable with their hands and that this attunement occurred only when the right hand was trained. Yet, substantial individual differences in attunement and the transfer thereof were found.

In what follows, we explore the implications of the present results for theories on the organization of perceptual learning and recent discussions on individual differences.

## Is attunement functionally organized?

The present study was based on the Gibsonian idea that animals can detect the same information in multiple ways. Just as an animal can achieve a motor function in multiple ways (Hebb, 1949; Lashley, 1930), perceivers, Gibson (1966) asserted, can detect the same informational structure in different ways, involving different anatomical structures (Bongers & Michaels, 2008; Oudejans et al., 1999). As mentioned in the *Introduction*, in the field of dynamic touch this multiple realizability of functions has been frequently demonstrated (Carello et al., 1992; Hajnal et al., 2007; Pagano et al., 1993). Hence, this makes dynamic touch a proper paradigm to study transfer of attunement. Is attunement confined to the limb that is trained to perceive length or does it transfer to the other limbs that can achieve this function?

We found that attunement is neither anatomically specific nor does it follow a functional organization. Simply looking at the improvements in length perception at the level of the group, a clear picture emerged: Training one of the hands resulted in an improvement with the hands but not with the feet. And training one of the feet gave rise to a smaller improvement in length perception, but one that was apparent in all the limbs. Examining the changes in what mechanical variables are exploited, and thus whether genuine attunement had occurred, led to a more complicated picture. Yet, asymmetrical transfer of attunement was still observed. In line with Hajnal et al. (2007), we found that in the absence of feedback, perceivers tended to rely on a nonspecifying variable with both their feet and their hands. That is, both in the vertical wielding condition (as used in the study of Hajnal et al., 2007) and in the horizontal wielding condition (as used in the present experiment), perceivers relied on mechanical variables that are not specific to length. However, after feedback differences between the anatomical structures emerged. Only when the right hand was trained, participants generally succeeded in detecting the specifying mechanical variable. The detection of this variable was apparent in the judgments with both hands but not with the feet. Indeed, participants continued to rely on nonspecifying variables with their feet. In contrast, training the left hand and the right foot did not result in attunement to the specifying variable. Yet the participants did not continue to rely on nonspecifying variables with any of their effectors when the left hand or the right foot were trained. After training the left foot, participants maintained reliance on a nonspecifying variable with this limb. However, they did not continue to rely on the variable they detected in the pretest with any of the other limbs. Hence, although attunement is not confined to the trained limb, the transfer does not follow a genuine functional organization either.

The big challenge now is how to account for these effects. One might argue that an explanation should be sought in the anatomical differences between hand and foot. The receptors in the hand and foot differ in their morphological and physiological characteristics (Kennedy & Inglis, 2002), explaining their differential sensitivity to, for example, temperature, vibrations, and touch (Hajnal et al., 2007, for an overview). Moreover, the rods were presented differently to the hands and to the feet—participants held the rod in their hand, whereas the rod was attached to a relatively heavy sandal (0.81 kg) when the length was to be judged with the feet. Hence, the receptors also were differently activated in the trials with the hands than in the trials with the feet.

Although the differences between the receptor characteristics of the hands and the feet are substantial, we consider it unlikely that these differences can account for the observed effects. First, the studies to date demonstrated that perceivers are capable of detecting the mechanical variables involved in dynamic touch with both effectors (Hajnal et al., 2007; Stephen & Hajnal, 2011), and the present study provides further evidence for this. Indeed, we found that for each limb the length perception of the vast majority of participants was reliably constrained by the nonspecifying mechanical invariants, at least in the pretest. Hence, with both their hands and feet perceivers are capable of extracting the mechanical invariants in dynamic touch, irrespective of the different morphology and physiology of their receptors, and irrespective of how the receptors are stimulated (i.e., holding the rod in the hand, or attaching it to a relatively heavy sandal). Second, the anatomical differences between the bodily parts cannot account for the observed training effects. We found that participants learned to rely on a specifying mechanical variable with both hands, but only when the right hand was trained, not when feedback was provided on the judgments with the left hand.

A more likely candidate for explaining the observed effects is to be sought in the wielding behaviour. Several studies have shown that the detection of a mechanical invariant in dynamic touch is reflected in the type of wielding behaviour (Arzamarski et al., 2010; Riley et al., 2002). More precisely, it has been suggested that by a certain wielding behaviour a particular mechanical invariant is extracted from the haptic array. Importantly, the differences in exploratory behaviour have not only been observed when different properties were to be perceived (Arzamarski et al., 2010; Riley et al., 2002), but also when participants learned to attune to a more useful informational variable to perceive a certain property (Arzamarski et al., 2010; Michaels et al., 2008). Michaels and Isenhower (2011), for example, found that in learning to perceive the partial length of rods by dynamic touch, the changes in what information was detected were accompanied by changes in the angular acceleration of the rod.

In the context of our study, this demonstrated relationship between exploratory behaviour and the detected information leads to several hypotheses that await further experimentation. First, it would suggest that participants generally did not succeed in learning to detect the specifying variable with their feet, because they did not master the required exploratory behaviour with this effector. Hence, the wielding behaviour with the feet should be different than the wielding behaviour with the hands that were successful in detecting the specifying information. Second, it would suggest that the dominant hand is the most successful limb in mastering the required exploratory skill and that this skill transfers to the nondominant hand but not to the feet. After all, only when the right hand was trained (and this hand was dominant in all except 3 participants) did the participants generally succeed in exploiting the specifying variable with the hands. Third, the demonstrated coupling of exploratory movements and the detection of information provides a new perspective on the observed individual differences in perceptual skills, a topic to which we shall now turn.

## Individual differences

There is a recent upsurge of studies on individual differences in perception and action (Arzamarski et al., 2010; Bergmann Triest & Kappers, 2007; Golenia, Schoemaker, Mouton, & Bongers, 2014; Jacobs, Michaels, & Runeson 2000; Kappers, 2003; Kostrubiec, Zanone, Fuchs, & Kelso, 2012; Michaels & de Vries, 1998; Michaels & Isenhower, 2011; Runeson & Andersson, 2007; Runeson, Juslin, & Olsson, 2000; Vegter, Lamoth, de Groot, Veeger, & van der Woude, 2014a; Vegter, de Groot, Lamoth, Veeger, & van der Woude, 2014b). Several studies have addressed the abundant variability within our species and did not follow the widely adopted method of averaging over participants and drawing general conclusions about human beings. During the past decade, individual differences in dynamic touch have been studied extensively (Arzamarski et al., 2010; Michaels et al., 2008; Stephen et al., 2010; Withagen & Caljouw, 2011; Withagen & Michaels, 2005). Using a relatively large sample size, Withagen and van Wermeskerken (2009), for example, observed that participants vary in how they respond to feedback and suggested that perceivers vary in their perceptual learning capacities (Menger & Withagen, 2009; Rop & Withagen, 2014).

The present study provides further evidence for individual differences in perceptual skills. For instance, although the analyses at the level of the groups revealed that participants succeeded in detecting a specifying variable with their hands when their right hand was trained, the analyses at the level of the individuals showed substantial individual differences. Only 5 of 15 participants learned to exploit a specifying variable. Moreover, although the analyses of the groups led to the conclusion that the participants did not succeed in detecting the specifying variable with their feet, one participant succeeded in doing so after the right foot was trained. Apparently, participants vary in their capacity to learn to detect specifying information with their different limbs (Menger & Withagen, 2009; Rop & Withagen, 2014; Withagen & van Wermerskerken, 2009).

In addition, the present study revealed individual differences in the transfer of attunement. In the group who received feedback on the right hand, only one of the five participants who detected a specifying variable with the right hand did so with his left hand in the posttest. Hence, although the analyses of the group might reveal that training the right hand resulted in the detection of a specifying variable with both hands, individuals vary in whether they do so. Moreover, we also observed that for *some* participants, training an effector did not result in an improvement in length judgments with that limb but led to attunement to a specifying variable with one of the other limbs. Hence, this provides even more compelling evidence that attunement is not organized in one way. Whether attunement occurs and in which limb it is manifested depends on the individual who is trained.

The theory that the wielding behaviour and the detection of mechanical invariants in dynamic touch are tightly coupled suggests that the above individual differences in the detection of information should manifest themselves in the individuals’ exploratory behaviour. Moreover, Stephen and Hajnal (2011) recently suggested that the fractality of the wielding behaviour can predict the amount of transfer of perceptual competence. In their study, they induced a recalibration in length perception with either the right hand or the right foot and observed an asymmetry in transfer—providing feedback on the foot recalibrated the foot more than it did the hand, whereas the foot and hand were equally recalibrated when the hand was trained. The analyses of the exploratory behaviour led them to conclude that the “temporal fractality” of the wielding behaviour can account for this effect. This sets a promising new line of research. Indeed, further studies are needed to test whether the nature of the wielding behaviour also can account for the amount of transfer of attunement and individual differences therein.

## Appendix

**Table 10:** Geometric and mechanical properties of the rods used in the experiment

Material	Length (m)	Diameter (m)	*m* (kg)	*M* (kg.m)	*I*_*1*_ (kg.m^2^)
Test rod set					
Carbon	0.71	0.020	0.093	0.033	0.016
Carbon	0.91	0.020	0.119	0.054	0.033
Carbon	1.01	0.020	0.132	0.067	0.045
Carbon	1.11	0.020	0.145	0.081	0.060
Carbon	1.21	0.020	0.158	0.096	0.077
Aluminum	0.91	0.016	0.494	0.225	0.136
Steel	0.61	0.012	0.524	0.160	0.065
Steel	0.71	0.012	0.610	0.217	0.102
Wood	0.81	0.012	0.064	0.026	0.014
Wood	1.01	0.012	0.080	0.040	0.027
Steel	0.61	0.008	0.232	0.071	0.029
Steel	0.71	0.008	0.271	0.096	0.046
Steel	0.81	0.008	0.309	0.125	0.068
Feedback rod set					
Steel	0.50	0.010	0.298	0.075	0.025
Steel	0.60	0.010	0.370	0.111	0.044
Steel	0.70	0.010	0.418	0.146	0.068
Steel	0.46	0.012	0.395	0.091	0.028
Steel	0.56	0.012	0.481	0.135	0.050
Aluminum	0.60	0.010	0.127	0.038	0.015
Wood	0.70	0.012	0.052	0.018	0.008
Wood	0.86	0.012	0.068	0.029	0.017
Aluminum	0.80	0.016	0.438	0.175	0.094
Carbon	0.60	0.020	0.066	0.020	0.008
Carbon	0.76	0.020	0.099	0.038	0.019
Carbon	0.86	0.020	0.105	0.042	0.022
Carbon	0.96	0.020	0.125	0.060	0.038

## Footnotes

1 All averaging and statistical tests done on correlations used the correlations’ *z*-transformations.
